# Cytokines as Mediators of Pain-Related Process in Breast Cancer

**DOI:** 10.1155/2015/129034

**Published:** 2015-11-09

**Authors:** Carolina Panis, Wander Rogério Pavanelli

**Affiliations:** ^1^Laboratory of Inflammatory Mediators, State University of West Paraná, UNIOESTE, Campus Francisco Beltrão, Rua Maringá 1200, 85605-010 Francisco Beltrão, PR, Brazil; ^2^Laboratory of Immunoparasitology, State University of Londrina, UEL, PR, Brazil

## Abstract

Pain is a clinical sign of inflammation found in a wide variety of chronic pathologies, including cancer. The occurrence of pain in patients carrying breast tumors is reported and is associated with aspects concerning disease spreading, treatment, and surgical intervention. The persistence of pain in patients submitted to breast surgery is estimated in a range from 21% to 55% and may affect patients before and after surgery. Beyond the physical compression exerted by the metastatic mass expansion and tissue injury found in breast cancer, inflammatory components that are significantly produced by the host-tumor interaction can significantly contribute to the generation of pain. In this context, cytokines have been studied aiming to establish a cause-effect relationship in cancer pain-related syndromes, especially the proinflammatory ones. Few reports have investigated the relationship between pain and cytokines in women carrying advanced breast cancer. In this scenario, the present review analyzes the main cytokines produced in breast cancer and discusses the evidences from literature regarding its role in specific clinical features related with this pathology.

## 1. The Cytokine Status in Breast Cancer: An Overview

Malignant solid tumors can metastasize and infiltrate important organs, damaging tissue and nervous structures, which causes pain. Breast cancer is a malignant cancer that frequently spreads and infiltrates distant sites in the body as the liver, lungs, brain, and bones. Therefore, organ infiltration by breast metastasis causes severe pain by multiple mechanisms [1, 2].

Host-tumor interactions favor the arising of several soluble mediators, including cytokines and chemokines. Although such molecules are not exclusive in breast cancer (as well its clinically related processes), there is a massive production of circulating cytokines during disease development that are likely enrolled in the systemic pain experienced by patients. Depending on the staging of disease, women with breast cancer exhibit distinct patterns of circulating cytokines. In the early stages, when breast cancer is localized, patients display reduced TNF-*α* and IL-12 in serum [3], and concomitantly there are few reports of pain. On the other hand, patients presenting advanced disease have high systemic levels of TNF-*α* and IL-1*β* [3], which are often accompanied by pain. These facts suggest a possible connection between cytokine patterns and pain in breast cancer.

Breast cancer is a heterogeneous disease and is divided into 4 main subtypes according to its clinical molecular characteristics as luminal A and luminal B and HER-2 amplified and triple negative tumors. Each subtype harbors specific clinical behavior and aggressiveness, which affect disease prognosis [4]. Luminal tumors present positivity to estrogen and/or progesterone receptors (luminal A) and can further present the amplification of the receptor of the human epidermal growth factor 2 (HER2). The latter is categorized as luminal B and is more aggressive than luminal A cancers. Some tumors present only the overexpression of HER2 and are named as HER2-amplified. Finally, tumors that do not exhibit any of these receptors are classified as triple negative [5]. This status is a determining factor not only for the clinical outcome of the patient presents but also for the type of circulating cytokines presented.

In this way, women bearing luminal tumors display higher TNF-*α* and TGF-*β*1 plasmatic levels than healthy women, and concomitantly reduced levels of IL-12. In relation to the other subtypes, patients with luminal tumors exhibit less TGF-*β*1 than those with HER2-amplified tumors, showing no further differences regarding TNF-*α*, IL-12, IL-1*β*, and IL-10. Patients diagnosed with HER2-amplified tumors present augmented TNF-*α* and TGF-*β*1 when compared to healthy individuals and sustain significantly higher levels of IL-12 in relation to the other subtypes. On contrary, triple negative patients present reduced circulating levels of TNF-*α* and TGF-*β*1 in relation to the other subtypes, as well as when compared to healthy volunteers [6]. Therefore, the systemic cytokine profile is closely related with tumor subtype and may affect disease outcome in some instance.

Chemotherapy can also modulate cytokine patterns during breast cancer treatment. A comparative analysis of cytokine levels between untreated patients and women ongoing adjuvant regimen (anthracycline-taxane based) shows that doxorubicin infusion can acutely downregulate TNF-*α* and IL-1*β* in plasma, while paclitaxel upregulates the circulating IL-10 after its 1-hour infusion. Moreover, direct exposure of blood cells to chemotherapy can lead to cytokine deregulation, as observed in healthy volunteers. Paclitaxel can reduce TNF-*α* levels after 1-hour direct contact with whole blood, and doxorubicin promotes reduction of IL-10 and simultaneous augment of IL-1*β* [7]. The fast capacity of chemotherapy to modulate cytokine availability suggests that such drugs may affect the cellular capability of releasing preformed cytokines, as well as regulating the dynamic of cytokine consumption by cells.

## 2. Cytokines and Pain in Human Breast Cancer

Although a wide variety of cytokines has been reported in breast cancer aspects, few studies have focused on understanding its relationship with pain. The persistent inflammatory status of cancer results in the production of a wide of cytokines and chemokines that act on the nociceptors causing hypernociception. In general, proinflammatory cytokines are directly related with pain generation, while the anti-inflammatory set constitutes a negative modulator of hypernociception [8, 9]. The role of cytokines in breast cancer is far to be understood; however, some evidences have emerged in the past years. Here we present information regarding the role of cytokines in specific clinical aspects of human breast cancer, as summarized in Table 1.

### 2.1. Bone Metastasis and Tumor Osteopathy

About 70% of women with advanced breast cancer may develop bone metastasis, a late complication of disease [10]. Such metastases are osteolytic and cause bone destruction, pain, and nerve compression syndromes. Bone metastasis accounts for most of breast-cancer derived metastases and involves a wide of inflammatory mediators, including cytokines and chemokines.

Among cytokines, TGF-*β* is one of the most abundant growth factors physiologically present in the bone matrix and is released during bone resorption. This mediator is an important regulator of osteoclast activity in homeostasis [11] and possesses functional effects related with the generation of pain in some models of bone-related diseases. Studies have pointed that during bone damage TGF-*β* is released by cartilage cells and mediates the expression of nerve growth factor (NGF) in chondrocytes, which could potentially be a source of pain under noninflammatory conditions [12]. Further, the functioning of TGF-*β* axis in bones can be suppressed by proinflammatory cytokines as IL-1*β* and TNF-*α* [13], commonly found in breast cancer. Therefore, TGF-*β* may act as a noninflammatory mediator of pain in bone-related pathologies.

In breast cancer, bone metastasis involves a tight interaction between tumor metastasis and host cells. It is estimated that this event starts when metastatic cells detach from the primary tumor, enter the bloodstream, and infiltrate the distal bone sinusoids and bone marrow. The adaptation of infiltrating metastatic cells to the bone environment allows its growing and invasiveness [14], resulting eventually in bone breaks and pain.

There is a feed-forward vicious cycle for TGF-*β* in breast cancer metastases to bones [15]. It has been proposed that breast metastatisation to the bones includes a sequential pattern of gene expression that mainly includes TGF-*β* signaling pathway [16], where tumors cells stimulate osteoclasts to bone matrix resorption, which releases growth factors that cyclically activates tumor cells [17] (Chirgwin and Guise, 2000).

TGF-*β* is further implicated in the pathogenesis of the osteolytic bone metastasis by inducing the production of parathyroid hormone-related protein, a major mediator of the osteolytic process [18]. Tumor-specific prometastatic T cells also contribute to bone lesions by activating the RANKL pathway after bone colonization [19].

Biphosphonates are a pharmacological class used for treating breast cancer patients presenting with bone metastasis, aiming to reduce the pathological osteolysis [20]. The mechanism of action of biphosphonates consists in inhibiting bone resorption and osteoclasts activity but also involves the induction of inflammation. In this way, biphosphonates are important to control the bone-related pain in advanced breast cancer, as well as the interaction of metastatic cells with bone components.

The relationship between pain and cytokines in the context of biphosphonates is not clear in human studies, but there are some evidences pointing a role for cytokines in the mechanism of action of these drugs. Kaiser et al. [21] demonstrated that biphosphonates can reduce the capacity of breast cancer metastasis to interact with extracellular matrix components and affects the secretion of the chemokine CCL2 by osteoblasts. Moreover, the proinflammatory status induced by biphosphonates is mediated by innate response components, as neutrophils, monocytes, MyD88-TLR4 signaling, and IL-1 [22]. These mediators may support in part the antiosteolytic effects of biphosphonates, which results in reduction of pain.

Therefore, immune-related processes that are linked to the bone destruction may contribute to the generation of pain symptoms in metastatic breast cancer; thus bone-driven blocking of TGF-*β* may be an interesting approach to avoid pain syndromes in advanced breast cancer.

### 2.2. Persistent Pain and Breast Surgery

Chronic pain is a common feature in patients with breast cancer [30] and is mainly related with the releasing of inflammatory mediators from the tumor, nerve involvement, and tissue injury. This process results from a network formed by proinflammatory cytokines, genes from the inflammatory pathways and cytokine genes polymorphisms [23].

In this context, a role for polymorphisms in cytokine-related genes and the individual susceptibility to pain in cancer has been discussed [31]. McCann and coworkers [23] investigated a group of patients with breast cancer prior to breast cancer surgery to evaluate the profile of genetic polymorphisms in inflammatory genes and its relation with the occurrence of pain. The authors found that about 25% of women experienced pain, reaching a significant pain score that affected their quality of life. Further, genetic variations enrolling IL1R1 (rs2110726) and IL13 genes were correlated with preoperative pain in breast cancer. Patients carrying the minor allele for a single nucleotide polymorphism (SNP) in IL1R reported less pain before breast surgery, while those with the minor allele for a SNP in IL13 reported significant pain prior to breast surgery. This study reinforces the relevance of cytokines in breast cancer pain independent on mechanical features or tissue damage. Experimental IL1 silencing decreases pain-related behaviors [32], while IL13 has anti-inflammatory activity and affects IL1 releasing [33]. Thus, these cytokines may represent important regulatory points in cancer pain controlling, independent of surgical intervention.

The persistence of pain after breast tumor removal is a problem of clinical relevance that includes mechanical and inflammatory components. The chronic postsurgical pain is a consequence of sustained nociceptors activation and nerve damage [34]. The surgical manipulation of the site of surgery releases innumerous inflammatory mediators that induce peripheral sensitization; therefore, sustained stimulation may result in persistent pain.

The maintenance of pain after breast surgery involves immune mechanisms recently identified by a cytokine signature study [24]. In such study, one single nucleotide polymorphism for interleukin 1 receptor (ILR2, rs11674595) and one haplotype for IL-10 (haplotype A8) were associated with the occurrence of persistent breast pain after surgery [24]. ILR2 gene encodes the IL-1 type II receptor that possesses anti-inflammatory effects, while IL-10 down regulates the proinflammatory cascade. The results from this study suggest that the IL-10 haplotype A8 diminishes the risk for development of severe chronic pain, while the polymorphism of ILR2 could be associated with increased risk for the occurrence of severe persistent breast pain.

### 2.3. Chemotherapy and Peripheral Neuropathy

Chemotherapy-induced peripheral neuropathy (CIPN) is a neurotoxic adverse effect of chemotherapy which is frequently related with the interruption of cancer treatment and therapeutic failure [35]. According to the National Cancer Institute (NCI) it constitutes the main reason for interrupting cancer treatment, which strongly affects patient outcome during treatment [36].

Evidences have suggested a role for cytokine-axis in regulating the chemotherapy-related pain. Studies concerning the quality of life in patients with breast cancer ongoing chemotherapy-based clinical trials have demonstrated that the combined treatments for metastatic breast cancer may induce short-lived episodes of pain [37, 38]. Early studies have established that doxorubicin-paclitaxel-based chemotherapy is a causative agent of pain in breast cancer and include mild peripheral neuropathy and mild myalgia/arthralgia episodes [39] (Amadori et al., 1996), but paclitaxel seems to be the main responsible for pain-related episodes in cancer chemotherapy.

Paclitaxel is one of the main drugs used for treating breast cancer. Its mechanism of action consists in affecting microtubule dynamics, inducing cell death. The same mechanism responsible for its antineoplasic effect is associated with its neurotoxic property. Paclitaxel may affect the peripheral nervous system by damaging microtubules and interfering in microtubule-based axonal transport, leading to peripheral nerve degeneration [35].

The direct effects of paclitaxel that cause peripheral neuropathy can be associated with the cumulative effect of subsequent doses of this drug. However, patients also experience acute episodes of neuropathy, which suggest that additional mechanisms may be included in the chemotherapy-induced neurotoxicity. Since cancer is an inflammatory disease, disruption of cytokines and inflammatory mediators may be enrolled in this process.

Experimental models of neuropathy suggest that blocking proinflammatory molecules can suppress the painful sensation [40], implicating cytokines as modulators of pain. Indeed, proinflammatory cytokines can modulate nociceptors [35].

Paclitaxel infusion can activate macrophages to infiltrate peripheral nerves and cause injury to sensory neurons [41]. The mechanisms of pain mediated by paclitaxel include the upregulation of chemokines as CCL3 [25], monocyte chemoattractant protein-1 (MCP-1), its cognate receptor CCR2 [26], and CX3CL1 [27]. Molecules related with innate immune response, as the toll-like receptor TLR4 and MyD88 signaling pathway, are also implicated [42]. In addition, joint pain has been reported in women with breast cancer undergoing paclitaxel weekly and correlates with increased IL-10 in plasma [28].

Thereby, cytokines have been pointed as putative targets for intervention in the chemotherapy-induced painful peripheral neuropathy. TNF-*α*, IL-1*β*, IL-6, and chemokines are highlighted as putative candidates for therapeutics against chemotherapy-induced peripheral neuropathy (CIPN), since they constitute a primary mechanism that allows the neuron-immune interfacing and are enrolled in pain and hyperalgesia mechanisms [43].

### 2.4. Estrogen Deprivation and Pain Syndromes

Postmenopausal women undergoing hormone replacement therapy possess reduced pain thresholds when compared to those without hormone replacement or men [44]. This fact suggests that ovarian-derived hormones may affect the individual susceptibility to pain. Estrogen deprivation can result in bone demineralization, osteoporosis, and bone fracture. Studies have reported that the estrogen-dependent bone resorption is regulated by upregulation cytokines as TNF-*α* [45] and other proinflammatory components as IL-1, IL-6, IL-17, and IFN-*γ* [46]. Younger age has been pointed as a risk factor for developing pain following breast cancer surgery [47], indicating a role for female hormones in breast cancer related-pain.

The use inhibition of estrogen production by using aromatase inhibitors has been associated with musculoskeletal pain in a mechanism enrolling proinflammatory cytokines and NF*κ*B modulation [48]. In spite of this, information regarding the direct relationship between cytokines and pain in cancer models of estrogen deprivation is unknown.

### 2.5. Pain and the Use of Granulocyte Colony-Stimulating Factor (G-CSF)

Several studies have reported the systemic effects of the granulocyte colony-stimulating factor (G-CSF) analog in breast cancer patients. G-CSF is a biologic response modifier, and its analog is widely employed in association with other antineoplastic drugs to revert the neutropenia resulting from chemotherapy. This association minimizes myelotoxicity, enhances neutrophil counts, and prevents delays in treatment [49].

In spite of its positive modulating effects on immune response of cancer patients, G-CSF-based treatments are known by causing significant pain-related symptoms. Bone, muscle, and joint pain are common side effects reported by breast cancer patients after G-CSF treatment, even after the modification of this drug to its pegylated form [50, 51]. The association of G-CSF with epirubicin for treating advanced breast cancer revealed that an important set of patients (55%) report myoskeletal pain [29].

These data point out the fact that the stimulation of granulopoiesis affects the peripheral sensitivity in patients with breast cancer and draw attention to the fact that the activation of immune response may be directly associated with triggering/enhancing pain sensation in patients undergoing chemotherapy.

## 3. Perspectives and Conclusions

Cytokines are clearly enrolled as mediators of pain in advanced breast cancer. TGF-*β* is the major mediator of the osteolytic lesions and bone-related pain. Some gene polymorphisms and haplotypes also appear to affect the individual susceptibility to pain sensitivity and involve IL1, IL10 and IL13 genes. Chemokines are mainly implicated in paclitaxel-mediated pain, while the G-CSF is a stimulator of muscular pain. Although few cytokines have been proved as direct effectors of pain in breast cancer, it is expected that the proinflammatory components as TNF-*α* and IL-6 may be enrolled in such process in some extent. Therefore, such mediators may constitute putative targets for pharmacological intervention aiming to control pain in women bearing advanced breast cancer.

## Figures and Tables

**Table 1:** 

Cytokine/chemokine	Role in breast-cancer-related pain	Reference
TGF-*β*	Major regulator of osteoclasts, affecting bone resorption and osteolysis	[15]
		[18]
		[17]

IL1R1 polymorphism	Patients carrying the minor allele for a single nucleotide polymorphism (SNP) in IL1R reported less pain before breast surgery	[23]

IL1R2 polymorphism	The SNP rs11674595 is associated with the occurrence of persistent breast pain after surgery	[24]

IL13 polymorphism	Women carrying the minor allele for a SNP in IL13 reported significant pain prior to breast surgery	[23]

IL10 gene haplotype	The haplotype A8 is associated with the occurrence of persistent breast pain after surgery	[24]

CCL3	Paclitaxel-mediated pain	[25]

CCR2	Paclitaxel-mediated pain	[26]

CX3CL1	Paclitaxel-mediated pain	[27]

IL10 (serum)	Paclitaxel-induced joint pain	[28]

G-CSF	Muscle pain	[29]
